# Encapsulation of *Mesona chinensis* Benth Extract in Alginate Beads Enhances the Stability and Antioxidant Activity of Polyphenols under Simulated Gastrointestinal Digestion

**DOI:** 10.3390/foods11152378

**Published:** 2022-08-08

**Authors:** Chonnipa Wongverawattanakul, Phim on Suklaew, Charoonsri Chusak, Sirichai Adisakwattana, Thavaree Thilavech

**Affiliations:** 1 Phytochemical and Functional Food Research Unit for Clinical Nutrition, Department of Nutrition and Dietetics, Faculty of Allied Health Sciences, Chulalongkorn University, Bangkok 10330, Thailand; 2 Department of Home Economics, Faculty of Agriculture, Kasetsart University, Bangkok 10900, Thailand; 3 Department of Food Chemistry, Faculty of Pharmacy, Mahidol University, Bangkok 10400, Thailand

**Keywords:** *Mesona chinensis*, alginate bead, encapsulation, antioxidant activity

## Abstract

The aim of this study was to investigate the stability and antioxidant activity of the polyphenols from *Mesona chinensis* Benth extract (MCE) and its alginate-based encapsulation by extrusion technique during simulated gastrointestinal digestion. The encapsulation efficacy ranged from 41.1 ± 4.7 to 56.7 ± 3.4% with different concentrations of MCE (50–75% *v*/*v*), sodium alginate (1.2–1.8% *w*/*v*), and CaCl_2_ solution (3–5% *w*/*v*). The optimal condition for MCE-loaded alginate beads (MCB) was composed of 75% MCE, 1.5% alginate, and 3% CaCl_2_ solution, which provided the highest encapsulation efficiency with a spherical structure and a mean particle diameter of 1516.67 ± 40.96 μm. Fourier transform infrared spectroscopy (FT-IR) reported no chemical interaction between alginate and MCE. The release of total phenolic content (TPC) was only 8.9% after placing MCB in water for 4 h. After simulated digestion, changes in TPC and ferric reducing antioxidant power (FRAP) of MCE significantly decreased by 25.0% and 29.7%, respectively. Interestingly, the incorporation of MCB significantly increased TPC and FRAP in the digesta compared to those of MCE during gastrointestinal tract conditions. The findings suggest that the encapsulation of MCE with alginate as a carrier helps to improve the bioaccessibility and biological activity of *M. chinensis* polyphenols.

## 1. Introduction

*Mesona chinensis* Benth (*Chinese Mesona*) is an economically important herbal plant belonging to the Lamiaceae family. It is widely cultivated in Southeast Asia and China. In addition to consuming it as a herbal drink and as grass jelly (a gelatin-type dessert), *M. chinensis* has been used as a traditional medicine to treat hypertension, diabetes, and liver diseases [1]. The extract of *M. chinensis* leaf has been reported to contain polyphenols with kaempferol and caffeic acid as the major bioactive compounds [2]. Due to the high polyphenol content, *M. chinensis* possesses antioxidant, anti-inflammatory, antiglycation, and antihyperglycemic activities [2,3,4,5]. The consumption of *M. chinensis* extract helps to improve plasma antioxidant status and attenuate postprandial glucose following a high-carbohydrate meal in overweight participants [6]. The inhibitory activity of *M. chinensis* extract against carbohydrate digestive enzymes, particularly intestinal α-glucosidase, maltase, and sucrase, was linked to its polyphenol content [6]. According to the pharmacological properties, *M. chinensis* becomes an interesting active ingredient for developing polyphenol-enriched food products. However, one of the major limiting factors affecting the beneficial effects of polyphenols is their bioaccessibility and bioavailability throughout the gastrointestinal tract, which may affect the achievable desired concentration at the site of action [7,8]. The improvement of bioaccessibility might increase the content of polyphenols in the digestive tract, which can potentially be absorbed or bioavailable to the body.

According to the self-assembly and gel-forming ability, polysaccharides have been used as coating agents to improve the bioaccessibility of polyphenols by controlling their release as well as preventing their degradation and delaying absorption under gastrointestinal tract conditions [9]. As a food-grade ingredient derived from brown seaweed, sodium alginate is a common coating material for encapsulation by the cross-linking process in the presence of divalent ions, commonly Ca^2+^, to trap the bioactive compounds inside [10]. This structure can tolerate an acidic environment and break down to promote the release of core compounds in an alkaline environment [10]. Interestingly, the encapsulation of plant polyphenols with calcium–alginate by the simple extrusion dripping technique has been shown to control the release of bioactive compounds into the small intestine [9]. For example, the alginate-based encapsulation of plant extracts, such as *Clitoria ternatea* and curcumin, increased the stability and bioactivity of their polyphenols after stimulated digestion [11,12]. In addition, the encapsulation of *Hypericum perforatum* also improved the thermal stability of the plant’s flavonoids [13]. However, there is no available data on the bioaccessibility of *M. chinensis* during gastrointestinal digestion. Therefore, it is worthwhile to evaluate the polyphenol stability of *M. chinensis* together with the development of its alginate-based encapsulation for food application. Furthermore, the digestive stability and biological activity of *M. chinensis* water extract (MCE) and its encapsulation in simulated digestion fluid were also investigated. Finally, the physical properties of MCE encapsulation with respect to the efficiency percentage, particle size distribution, microstructure, and melting temperature were examined.

## 2. Materials and Methods

### 2.1. Materials

Folin–Ciocalteau’s reagent, 2,4,6-Tris(2-pyridyl)-s-triazine (TPTZ), porcine pepsin from gastric mucosa powder, α-amylase Type VI-B from porcine pancreas, pancreatin from porcine pancreas, and gallic acid were purchased from Sigma (St. Louis, MO, USA). Amyloglucosidase from *Aspergillus niger* was purchased from Roche Diagnostics (Indianapolis, IN, USA). Sodium alginate (food grade) and calcium chloride (food grade; CaCl_2_) were obtained from Nerdy gummy (Bangkok, Thailand).

### 2.2. Plant Materials

Dried stems and leaves of *M. chinensis* were purchased from a local market in Thailand. The plant was authenticated at the Professor Kasin Suvatabhandhu Herbarium, Department of Botany, Faculty of Sciences, Chulalongkorn University, Thailand, Voucher specimen: A013637 (BCU). The *M. chinensis* water extract (MCE) was obtained based on a previous study with some modifications [6]. The dried *M. chinensis* leaves (600 g) were boiled in distilled water (4 L) at 90 °C for 4 h and then left at 60 °C for another 4 h to evaporate the solvent. The extract was then sieved through cheesecloth, filtered through Whatman paper No. 4 and No. 1, and stored at −20 °C in darkness for further analysis.

### 2.3. Preparation of Alginate-Based Encapsulation of MCE

The encapsulation process was conducted based on a previous study [11]. Sodium alginate (1.2, 1.5, and 1.8% *w*/*v*) was dissolved in warm distilled water (approximately 70 °C). The MCE (50 and 75% *v*/*v*) was further added and mixed using a magnetic stirrer for 5 min to yield a homogenous solution. To develop *M. chinensis* beads (MCB), the mixture was loaded into the syringe with a stainless needle (0.5 × 25 mm) and sonicated for 15 min to remove air bubbles before being added dropwise to CaCl_2_ solution (3 and 5% *w*/*v*) by the syringe pump (Aitecs, Vilnius, Lithuania). The dropping rate and distance between the needle and the solution surface were fixed at 15 mL/h and 3 cm, respectively. The calcium-alginate bead without MCE was performed as a control bead. After 15 min of curation, the beads were washed with distilled water, air-dried overnight at room temperature, and kept in a desiccator at room temperature for further analysis.

### 2.4. Determination of Total Polyphenol Content (TPC)

TPC was measured by the Folin–Ciocalteau method with a slight modification [6]. MCE was dissolved in distilled water. The sample solution (50 µL) was mixed with 50 µL Folin–Ciocalteau reagent (10-fold dilution in distilled water) and incubated at room temperature. After 5 min, sodium carbonate (10% *w*/*v*; 50 µL) was added and incubated for 30 min. The absorbance was measured at 760 nm. TPC was expressed as mg gallic acid equivalent (GAE)/mL using a gallic acid (10–100 μg/mL) standard curve (r^2^ = 0.996).

### 2.5. Encapsulation Efficiency

All beads made from 2 mL of the mixture (MCE + alginate) in each condition were dissolved in 4 mL of sodium acetate (5% *w*/*v*), mixed until fully dissolved, and centrifuged at 906× *g* for 5 min. The disintegrated MCB supernatant was collected for the determination of TPC. The percentage encapsulation efficiency was calculated based on the following equation [11]:

Encapsulation efficiency (%) = (TPCb ÷ TPCe) × 100,(1)

where TPCe was the total polyphenol content in the MCE solution before the encapsulation, while TPCb was the total polyphenol content in the disintegrated MCB supernatant from the same concentration of the extract.

### 2.6. Morphology and Particle Size Analysis

The morphology and the surface appearance of beads were observed under a scanning electron microscope (SEM; JEOL Ltd., Tokyo, Japan) according to a previously published report [11]. The beads were prepared for SEM analysis by attaching them to a stub using a two-sided adhesive tape and coating them with gold (40 nm) as a conductive material using an ion sputter instrument (model SCD 040, Bal-Tec, Balzers, Liechtenstein). The coated sample was observed under SEM with a vacuum condition at 15 kV. The particle diameter of the beads was determined by a laser diffraction-based particle size analyzer (Malvern Instruments Inc., Worcestershire, UK). The median of the particle size distribution was calculated based on a refractive index of 1.52 for CaCl_2_, 1.37 for sodium alginate, and 1.38 for MCE [11].

### 2.7. Thermal Behavior

The thermal stability was examined by differential scanning calorimetry (DSC; Erich NETZSCH GmbH & Co. Holding KG, Selb, Germany). A dry sample (5 mg) was sealed in the pan and held at 25 °C for 1 min, then scanned from 25–350 °C with a heating rate of 10 °C/min. The flow rate of nitrogen purge gas was 50 mL/min. The melting temperature (T_m_) and crystallization temperature (T_c_) present in the thermograms were compared between each sample.

### 2.8. Fourier-Transform Infrared Spectroscopic (FTIR) Analysis

The interaction of MCE and sodium alginate in MCB was observed by using FT-IR with an attenuated total reflectance (ATR) accessory (Perkin Elmer, Norwalk, CT, USA). The FT-IR analysis was conducted according to the previous study [11]. The sample was compressed between two ART diamond crystals. Spectra of the samples were recorded in the transmission mode at a resolution of ±4 cm^−1^ and 32 scans, covering a wavenumber range of 400–4000 cm^−1^.

### 2.9. Simulated Gastrointestinal Digestion

The in vitro digestion of MCE and MCB was performed following a previous method by Pasukamonset et al. [11]. The amount of the sample for simulated digestion was adjusted according to TPC, which was 2 mg GAE/mL. The gastric phase of simulated digestion was initiated by mixing the sample with porcine pepsin solution (40 mg/mL in 0.1 N HCl) and adjusting pH to 2.0 ± 0.1 with 0.1 N HCl. The reaction mixture was incubated at 37 °C for 1 h in a shaking water bath (100 rpm) before adjusting the pH to 4.5 ± 0.1. The amyloglucosidase solution (0.125 mg/mL, 150 µL) was added and incubated for 30 min before adjusting pH to 5.3 ± 0.1 with a mixture of 1 N NaOH and 0.1 N NaHCO_3_. The small-intestinal enzyme solution containing pancreatin (3 mg/mL) and bile extract (12 mg/mL) in 0.1 N NaHCO_3_ was added and pH was adjusted to 7.2 ± 0.1, and the total volume was 20 mL in phosphate buffer saline. The digesta were collected after the gastric phase and at 0, 0.5, 1, 1.5, and 2 h of the intestinal phase. The reaction was terminated by centrifugation at 14,490× *g*, 4 °C and filtered through a 0.22 µm nylon filter. The TPC and antioxidant activity of digesta were analyzed immediately and reported as the percentage changes during simulated digestion relative to the amount of those in the gastric phase. 

### 2.10. Antioxidant Activity

A ferric reducing antioxidant power (FRAP) assay was conducted based on a previous study [11]. Briefly, the sample solution (20 µL) was incubated with 180 µL of FRAP reagent (containing 0.3 M sodium acetate buffer, pH 3.6, 10 mM TPTZ, and 20 mM FeCl_3_ at a ratio of 10:1:1) for 30 min. The reaction was centrifuged at 906× *g* at 4 °C for 1 min. The supernatant was collected for absorbance measurement at 593 nm. The results were expressed as mM FeSO_4_ equivalent/mL.

### 2.11. TPC Release

The in vitro release profile of MCB was studied according to a previous study with modification [14]. In brief, MCB (1 g) was left at room temperature in distilled water (1 mL) for 0, 0.5, 1, 2, 3, and 4 h. The TPC released in the dissolution medium was measured by the Folin–Ciocalteau assay as described previously.

### 2.12. Statistical Analysis

The results were expressed as mean ± SEM. All samples were conducted using three replications with three independent experiments. One-way ANOVA followed by Duncan’s post hoc test was evaluated for the significant differences among groups (*p* < 0.05). Two-way ANOVA followed by Duncan’s post hoc test was evaluated for the significant differences among groups and time points during simulated digestion. Statistical significance was defined as *p* < 0.05. All statistical analysis was conducted using SPSS version 21.0.

## 3. Results and Discussion

### 3.1. Total Polyphenol Content (TPC) and Antioxidant Activity of MCE

The results showed that the TPC of *M. chinensis* extract (MCE) was 24.20 ± 1.35 mg GAE/mL extract. The antioxidant activity of MCE presented by FRAP value was 18.80 ± 1.24 mM FeSO_4_/mL extract. The TPC of MCE in the present study exhibited antioxidant activity, which is in line with previous studies [6,15]. Chusak et al. reported that the TPC and FRAP value of aqueous *M. chinensis* dried extract were 212.37 ± 5.64 mg GAE equivalent/g dried extract and 1.42 ± 0.06 mM FeSO_4_ equivalent/mg dried extract, respectively [6]. In addition to aqueous extract, the large amounts of phenolic compounds and strong antioxidant activity of *M. chinensis* were observed when acidic ethyl acetate was used as the solvent extraction at pH 2 [15].

### 3.2. Encapsulation Efficiency and Characteristics of M. chinensis Beads (MCB)

#### 3.2.1. Encapsulation Efficiency of MCB

In a preliminary study, MCBs could not hold the spherical shape when the concentration of sodium alginate and CaCl_2_ was below 1.2% (*w*/*v*) and 3% (*w*/*v*), respectively. Therefore, the concentration of MCE at 50–75% (*v*/*v*), sodium alginate at 1.2–1.8% (*w*/*v*), and CaCl_2_ solution at 3–5% (*w*/*v*) were varied to perform 10 formulae of MCBs. As shown in Table 1, the percentage encapsulation efficiency (%EE) of MCBs ranged from 41.1 to 56.7%. No significant difference in %EE was observed for the different concentrations of CaCl_2_ (*p* > 0.05). According to the results, the alginate concentration was found to influence the spherical shape of MCBs. In the present study, the best concentration of alginate required to enable the formation of spherical beads was at 1.5% (*w*/*v*), while alginate solution at 1.8% (*w*/*v*) led to tear-shaped beads with tail structure due to the high viscosity of MCE–alginate solution dropped into CaCl_2_ solution (data not shown). According to the formation of alginate cross-link with Ca^2+^, low alginate concentration may cause a lack of COO^−^ group to interact with Ca^2+^, causing the deformation in bead shape [11,16]. On the other hand, the higher concentration of sodium alginate forms a highly viscous solution that increases the resistance to forming a spherical shape when the droplet falls [17]. This may be explained by the impact of alginate concentration on the surface tension of the solution which influences the shape of the calcium–alginate beads. When the concentration of alginate is increased, the surface tension of the alginate solution is lower than that of water, and the droplet is not able to form a perfect sphere prior to the gelation process in CaCl_2_ solution, resulting in a tear-drop shape of the beads [18]. 

Although no significant difference (*p* > 0.05) was obtained between the %EE of samples, the MCB composed of 75% (*v*/*v*) MCE, 1.5% (*w*/*v*) alginate, and 3% (*w*/*v*) CaCl_2_ with %EE of 56.7 ± 3.4% was selected for further experiments. The TPC of MCB was 169.31 μg GAE/mg, which was higher than that of rose hips (*Rosa canina*) alginate–chitosan beads (38.9 µg GAE/mg) [19]. 

#### 3.2.2. Morphology of MCB

**Figure 1:**
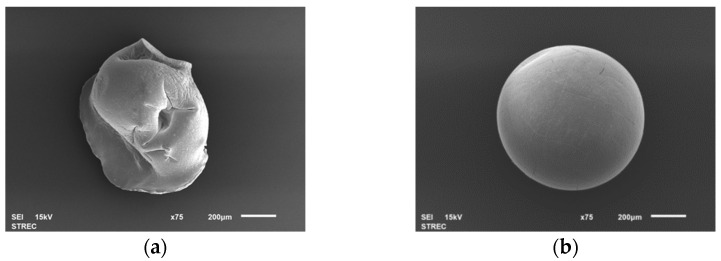
Morphology of (**a**) the control and (**b**) *Mesona chinensis* bead (MCB) under a scanning electron microscope (SEM).

The surface morphology of calcium alginate beads (control) and the selected MCBs were observed under SEM. The control bead showed a non-spherical shape (a shriveled shape) with some surface gaps (Figure 1a), while MCB had a spherical structure with less visible cracks (Figure 1b). The spherical shape of microbeads is formed due to the linkage of polyphenol and alginate [20]. The spherical shape of microbeads has been reported to have greater gel bead strength than non-spherical shapes [21].

#### 3.2.3. Particle Size of MCB

**Figure 2:**
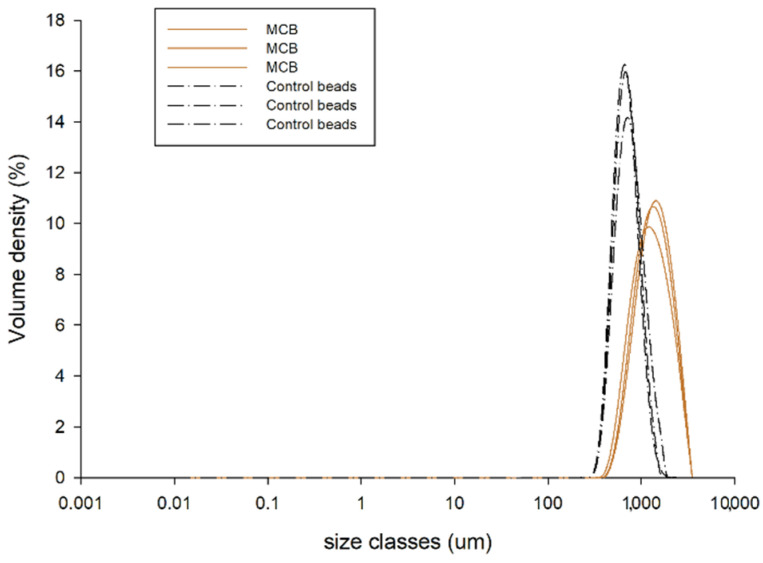
The particle size distribution obtained from the light-diffraction particle size analyzer of control beads and *Mesona chinensis* beads (MCB) (*n* = 3).

As shown in Figure 2, the beads loading MCE had a mean diameter of 1516.67 ± 40.96 μm, which was higher than that of the control beads (792.33 ± 26.12 μm). The results suggest that the diameter of MCB increased as the plant extract was trapped inside [11,12]. The simple injection method used in this study commonly produces beads with a diameter of greater than 1000 μm [18]. The size of alginate-based beads greatly varied due to many factors, such as the density, viscosity, and concentration of alginate, calcium, and active compounds, the type and diameter of dropping tools, and curing time [18].

#### 3.2.4. Thermal Profile of MCB

**Figure 3:**
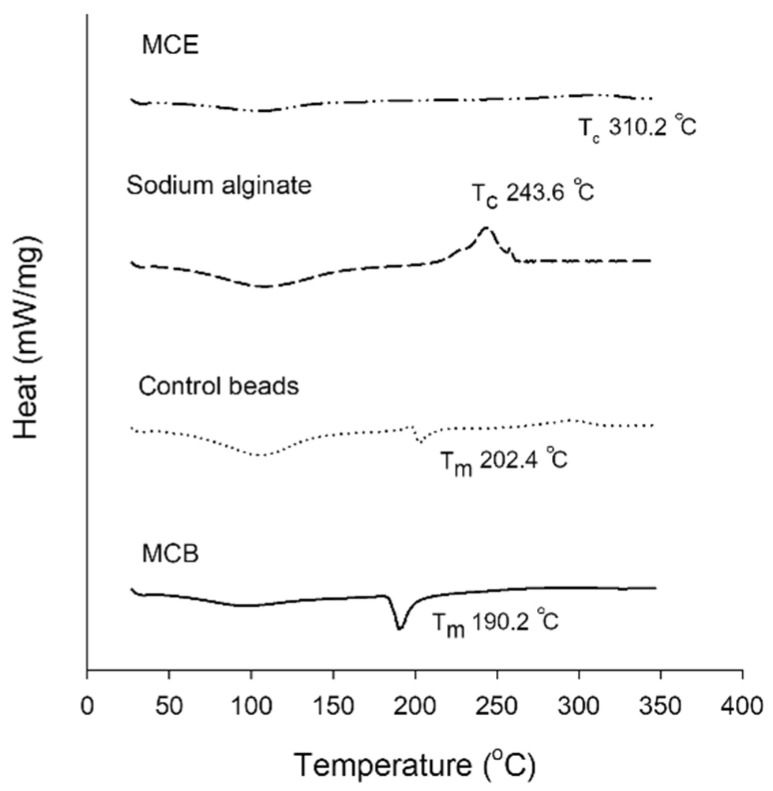
Thermograms by differential scanning calorimeter (DSC) for *Mesona chinensis* extract (MCE), sodium alginate, control beads, and *Mesona chinensis* beads (MCB).

The thermograms representing the thermal profiles of MCE, sodium alginate powder, control beads, and MCB are shown in Figure 3. Sodium alginate showed an exothermic decomposition peak at 243.6 °C, which was similar to the results of previous studies [11,22]. Control beads and MCE had broad exothermic peaks at 295.4 °C and 310.2 °C, respectively. The broad endothermic transition temperature for all samples was around 100 °C. However, in the present study, no sharp endothermic peak that corresponded to the melting curve of MCE was found. The absence of melting point was also found in lyophilized yerba mate extract, which may be explained by the diversity of compounds in plant extract [23]. The MCE incorporation into alginate beads caused the melting point to shift from 202.4 °C in the control bead to 190.2 °C in MCB. According to this result, encapsulation with alginate may help to improve the stability of MCE polyphenols to thermal degradation during food processing and cooking conditions [24].

#### 3.2.5. Chemical Interaction of MCB

**Figure 4:**
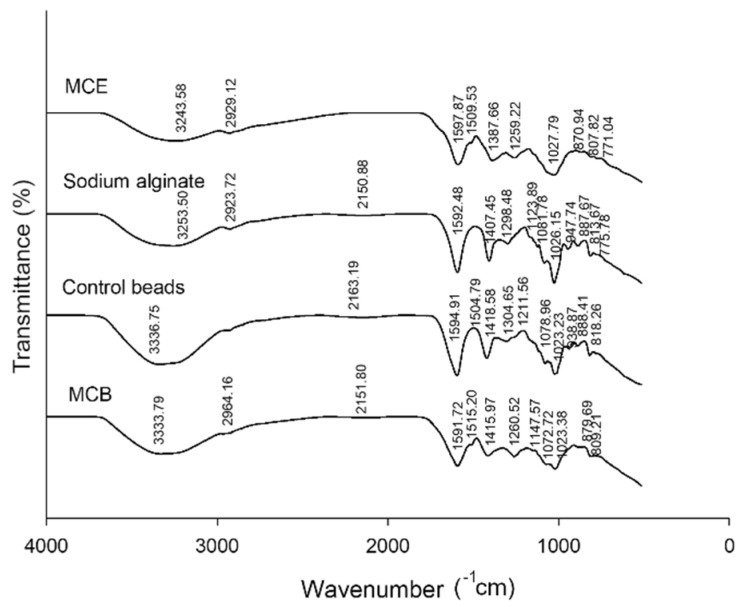
Fourier transform infrared spectroscopy (FT-IR) spectra of *Mesona chinensis* extract (MCE), sodium alginate, control beads, and *Mesona chinensis* beads (MCB).

The chemical interactions of MCE, sodium alginate, the control bead, and MCB were investigated using FT-IR spectroscopy (Figure 4). The FT-IR spectra of sodium alginate powder showed a major band of O-H stretching at 3253.50 cm^−1^, C-O-C stretching at 1026.15 cm^−1^, COO^−^ (asymmetric) at 1592.48 cm^−1^, and COO^−^ (symmetric) at 1407.45 cm^−1^. The strong and wide absorption band characteristic of O-H stretching in sodium alginate powder was similar to the previous studies [11,22]. Theoretically, the occurrence of cross-linking reactions between Ca^2+^ and alginic acid (G)-block or COO^−^ groups of alginate affects the intensity of the asymmetry and symmetry COO^−^ stretching bands [25]. After encapsulation, strong asymmetry COO^−^ stretching was observed at 1594.91 cm^−1^ for the control bead and at 1594.91 cm^−1^ for MCB, and a weak symmetric peak was presented at 1418.58 cm^−1^ and 1415.97 cm^−1^ on the spectra of control beads and MCB, respectively. The wavenumber of C-O-C and intensity was decreased in the control beads (1023.23 cm^−1^) and MCB (1023.38 cm^−1^) when compared to sodium alginate powder. These changes occurred due to the presence of an ionic bond between COO^−^ of alginate and Ca^2+^ [26].

In addition, a new peak was observed at 2151.80–2163.19 cm^−1^ in the control bead, indicating the crosslinking between calcium and alginate as suggested in the previous study [11]. The O-H stretching of both control beads and MCB shifted to a higher wavenumber and increased intensity. This may be explained by the intermolecular hydrogen bonds between plant extract and alginate [11,25]. In MCB, the intensity of the peak at 1260 cm^−1^ indicated that aromatic ring stretching of plant polyphenols was raised in the spectrum of alginate after incorporating with MCE [27]. However, this formation did not alter the new peaks after the MCB formation. The findings suggest that no chemical interaction was found between MCE and alginate. Therefore, the encapsulation with alginate as a coating material could be used as a carrier of MCE polyphenols for food or nutraceutical ingredients.

### 3.3. Kinetic Release of Polyphenols from MCB

**Figure 5:**
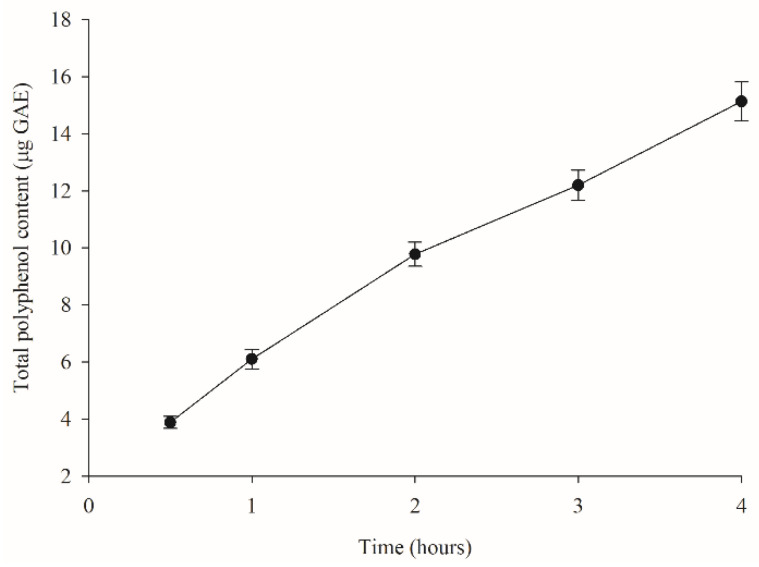
Total polyphenol content (TPC) released in distilled water from *Mesona chinensis* beads (MCB) at 0.5, 1, 2, 3, and 4 h. Data are expressed as mean ± SEM (*n* = 3).

The TPC release of MCB in distilled water was performed to simulate a controlled delivery system used in food products and beverages. TPC was used as an indicator to monitor the release of polyphenols from MCB in the dissolution medium. At 1 mg of MCB containing 169.31 μg GAE, the TPC released from the bead was 2.3, 3.1, 5.8, 7.2, and 8.9% at 0.5, 1, 2, 3, and 4 h, respectively (Figure 5). Zhang et al. reported the fast initial polyphenol release from calcium–alginate encapsulation of mulberry pomace by up to 50%, which may result from the rapid dissolution of phenolic compounds on the surface of the beads. For a longer period, the release rate slowed down as the release of phenolic compounds switched from the surface to the inside of the beads [14,28]. In the present study, almost 90% of MCE polyphenols were retained in the bead structure after 4 h. It could be related to the bonding, such as hydrogen bonds, between compounds and alginate [29], as supported by an increase in the O-H stretching intensity in MCB detected by FT-IR. 

### 3.4. The Polyphenolic Content and Antioxidant Activity of MCE and MCB after Simulated Digestion

In general, polyphenols are sensitive to many factors such as light, heat, pH, and temperature, leading to oxidation and epimerization [30,31]. The stability and release profile of polyphenols from MCE and MCB during simulated digestion were expressed as the percentage change of TPC released at 0, 0.5, 1, 1.5, and 2 h of intestinal phase from the amount of TPC released at the gastric phase (Table 2). The gastric TPC values were 103.35 ± 3.16 and 11.24 ± 0.58 mg GAE/mL digesta for MCE and MCB, respectively. The gastric FRAP values were 674.48 ± 27.92 and 74.74 ± 4.92 µg FeSO_4_ equivalent/mL digesta for MCE and MCB, respectively. The results showed that the MCE polyphenol content significantly decreased by 25.0% after simulated digestion. On the other hand, alginate-based encapsulation of MCE improved the bioaccessibility of polyphenols over time during in vitro digestion, and the TPC release reached its maximum within 1 h. At all time points, the released TPC from MCB was significantly higher than that of MCE. According to the results, the gradual increase of TPC release from MCB might be explained by the response of alginate to the environmental pH. At low pH in gastric conditions (pH 2), it was lower than the pKa value of mannuronic (pKa = 3.38) and glucuronic acid (pKa = 3.65), causing the calcium–alginate structure to form a stable hydrogen bond and retain the polyphenol content inside the beads. However, at pH higher than the alginate monomers’ pKa in the intestinal environment, the hydrogen bond network could be broken, leading to swelling of the alginate beads and consequently releasing the polyphenols [32]. Therefore, the encapsulation form of MCE polyphenols might improve the stability throughout the gastrointestinal tract and the chance of absorption in the intestine.

Polyphenols have been shown to be responsible for the antioxidant activity of plant extracts [29]. The percentage changes of antioxidant activity of MCE and MCB during digestion presented by FRAP value are shown in Table 2. The FRAP values of MCE markedly declined by 29.7% (*p* < 0.05), whereas the encapsulation of MCE with calcium–alginate significantly increased the FRAP value at all time points during simulated digestion. In this study, the improvement of polyphenol bioaccessibility may potentially increase their absorption, resulting in achieving systematic concentration and demonstrating antioxidant activity [7,8]. For example, the nano-encapsulation of curcumin enhanced serum total antioxidative capacity, which was superior to non-encapsulated curcumin in rats with myocardial infarction [33]. However, further study is needed to establish the bioavailability and biological activity of MCB under physiological conditions in the human body. 

## 4. Conclusions

The findings demonstrate that alginate-based encapsulation was able to increase the TPC and antioxidant activity of MCE after simulated gastrointestinal digestion. Therefore, the encapsulation of *M. chinensis* extract using alginate could be a technique to improve the bioaccessibility and biological activities of polyphenol.

## Figures and Tables

**Table 1:** The percentage encapsulation efficiency (%EE) of beads with different concentrations of *M. chinensis* extract (MCE), calcium chloride solution (CaCl_2_), and sodium alginate.

MCE (% *v*/*v*)	CaCl_2_ (% *w*/*v*)	Sodium Alginate (% *w*/*v*)	%EE
50	3	1.5	41.1 ± 4.7 ^a^
50	5	1.5	42.2 ± 5.0 ^a^
50	3	1.8	42.0 ± 6.1 ^a^
50	5	1.8	48.7 ± 4.3 ^ab^
75	3	1.2	52.6 ± 1.0 ^bc^
75	5	1.2	52.0 ± 2.4 ^bc^
75	3	1.5	56.7 ± 3.4 ^c^
75	5	1.5	55.7 ± 2.4 ^c^
75	3	1.8	53.4 ± 4.9 ^bc^
75	5	1.8	55.0 ± 1.8 ^c^

Data are expressed as mean ± SEM (*n* = 3). Different letters in the same column indicate a significant difference at *p* < 0.05.

**Table 2:** The percentage changes of total phenolic content (TPC) and the ferric reducing antioxidant power (FRAP) during simulated digestion.

Experiments	Intestinal Phase				
	0 h	0.5 h	1 h	1.5 h	2 h
	% Change of TPC from gastric phase				
MCE	−9.7 ± 3.9 ^aA^	−11.3 ± 4.3 ^aA^	−15.8 ± 4.0 ^abA^	−19.4 ± 4.4 ^abA^	−25.0 ± 4.1 ^bA^
MCB	203.7 ± 21.5 ^aB^	356.3 ± 64.0 ^bB^	572.5 ± 60.4 ^cB^	538.9 ± 106.9^cB^	575.6 ± 64.6 ^cB^
	% Change of FRAP from gastric phase				
MCE	−12.7 ± 1.7 ^aA^	−26.5 ± 2.6 ^bA^	−25.9 ± 2.3 ^bA^	−30.2 ± 3.6 ^bA^	−29.7 ± 3.4 ^bA^
MCB	76.3 ± 4.4 ^aB^	143.1 ± 14.4 ^abB^	193.0 ± 17.3 ^bcB^	233.7 ± 24.8 ^cB^	236.0 ± 26.6 ^cB^

MCE—*M. chinensis* extract, MCB—*M. chinensis* beads. Data are expressed as mean ± SEM (*n* = 3). Data were analyzed using two-way ANOVA followed by Duncan post-hoc test. Different letters indicate a significant difference at *p* < 0.05. Capital letters ^A–B^ represent the different comparisons between MCE and MCB at each time point, and noncapital letters ^a–c^ represent the different comparisons between 0–2 h time points for each sample.

## Data Availability

Data is contained within the article.
